# Lymphoma Presenting as Cancer of the Glans Penis: A Case Report

**DOI:** 10.1155/2012/948352

**Published:** 2012-09-29

**Authors:** Konstantinos Stamatiou, Nikolaos Pierris

**Affiliations:** Department of Urology, Tzaneio General Hospital, Afendouli 1 Avenue, 18536 Piraeus, Greece

## Abstract

Penile lymphoma is a very rare neoplasm. We report the case of an 82-year-old man who presented with phimosis. The patient also complained of frequent and painful urination. Upon examination a painless penile ulcer and multiple enlarged inguinal lymph nodes were found. The shaft of the penis and the prostate were hard on palpation. Abdominal and transrectal ultrasound examination confirmed the involvement of the penis shaft and the prostate and also revealed involvement of the urinary bladder. Biopsy showed diffuse, large B-cell lymphoma. The patient was treated with systemic chemotherapy with full remission of the disease. We review the literature relevant to penile lymphoma and discuss this uncommon condition.

## 1. Introduction

Penile malignant tumors are generally uncommon. The most common type, squamous carcinoma, accounts for less than 1% of all malignancies in men in the USA and for 0.1% of cancer deaths [1]. Although non-Hodgkin lymphoma occurs at extranodal sites in up to 48% of patients [2], lymphomas of the penis are rare and in most cases represent secondary involvement of the penis by lymphoma due to haematogenous or lymphatic spread or due to direct infiltration from a neighboring organ [3]. Thus, primary penile lymphoma is extremely rare and indeed less than 30 cases have been reported in the literature [4]. Given its rarity, it is unusual for physicians to consider lymphoma in the differential diagnosis of a penile mass.

## 2. Case Presentation 

**Figure 1:**
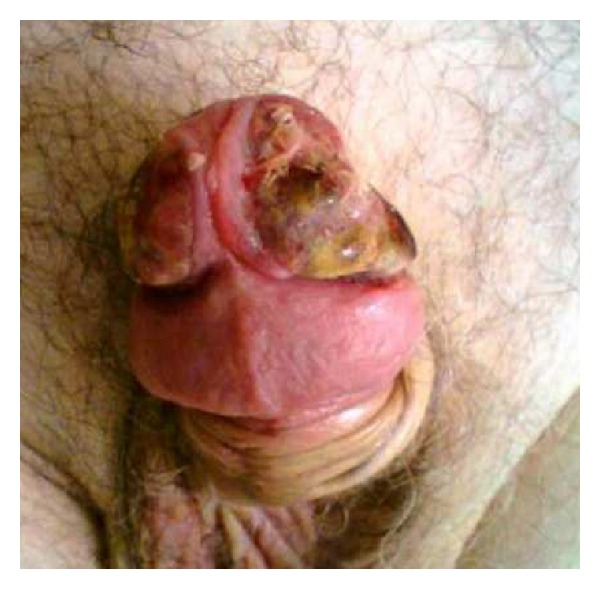
Dorsal view of the necrotic ulcer on the glans penis.

**Figure 2:**
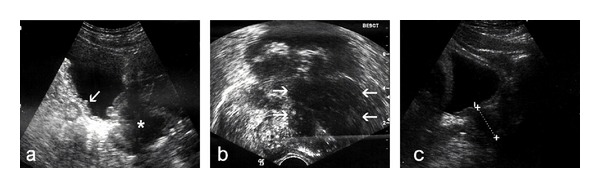
Sonographic imaging of the case. (a) Transabdominal US of the pelvis (sagittal section) shows an enlarged and markedly hypoechoic prostate (asterisk), with indistinct borders. Irregular thickening of the bladder wall is also noted (arrow). (b) Transrectal US confirms infiltration of the prostate by a bulky, lobulated, hypoechoic mass (arrows) which also involves the base of the penis. (c) Transabdominal US (comparable section with (a)) shows resolution of the infiltrative process.

**Figure 3:**
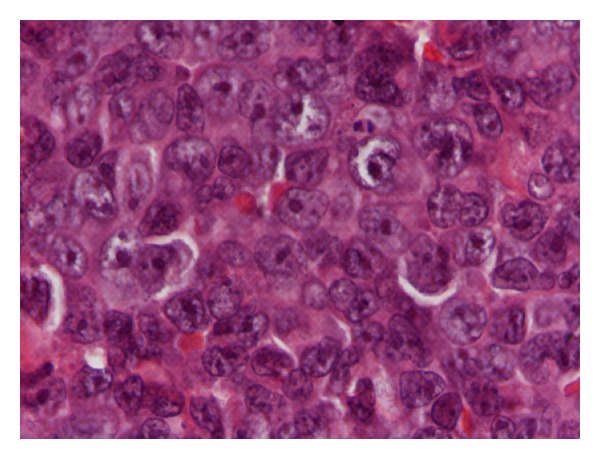
Pathology image.

**Figure 4:**
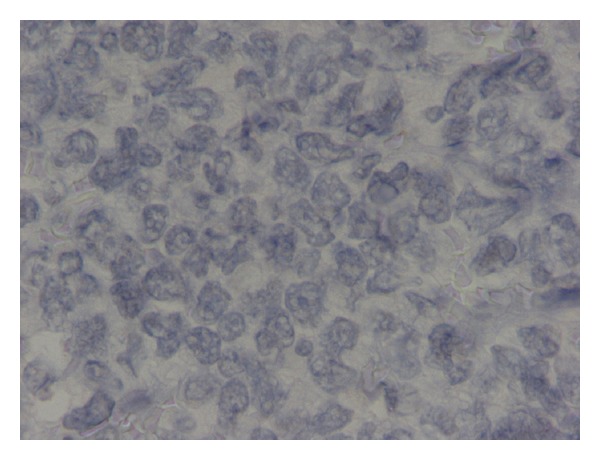
Pathology image (CD19 immunostain).

**Figure 5:**
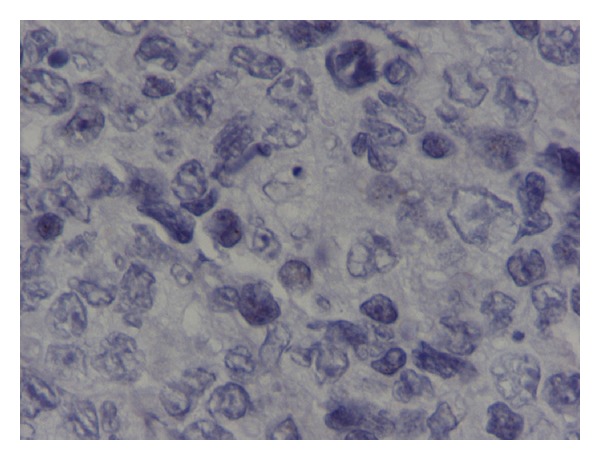
Pathology image (CD30 immunostain).

An 82-year-old man presented with phimosis. He also complained of frequent and painful urination. He had a history of chronic hepatitis C. His physical examination revealed a large well-demarcated penile ulcer with a necrotic base (Figure 1). The shaft of the penis and the prostate were hard on palpation. Multiple enlarged inguinal lymph nodes and mild abdominal tenderness were also noted. No urethral discharge, scrotum abnormalities, or hepatosplenomegaly were noted. Full blood count results, serum biochemistry tests, and serum electrolytes were normal. Serum PSA was 0.52 ng/mL. Abdominal ultrasound (US) showed a normal shape and configuration of both kidneys. Irregular thickening of the urinary bladder was noted. The prostate was enlarged (volume: 80 mL), hypoechoic and with indistinct borders (Figure 2(a)). Transrectal US examination showed that the shaft of the penis and the right prostate lobe were infiltrated by a solid, ill-defined, hypoechoic mass (Figure 2(b)). Multiple irregular solid masses were also found to protrude into the lumen of the bladder. Computed tomography (CT) of the abdomen and pelvis showed pelvic lymphadenopathy and significant prostate enlargement but failed to provide further information. CT of the chest showed no metastases to the lungs or thoracic lymphadenopathy. Magnetic resonance imaging (MRI) of the upper and lower abdomen showed a tumor in the location of the seminal vesicles and prostate of nodular/tubular inhomogeneous composition exhibiting little contrast enhancement, as well as thickening of the bladder wall and a filling defect between 5 o'clock and 9 o'clock, probably due to arteriovenous/lymphatic dysplasia. The remaining abdominal organs and lymph nodes revealed no pathological findings. Fine needle aspiration of one of the inguinal masses and biopsy of the penile ulcer and bladder tumor were performed. The histopathologic examination showed that the tissue samples from the penis were covered focally by stratified squamous epithelium and exhibited surface ulceration. The underlying layers were invaded to a considerable extent by a malignant neoplasm of lymphatic origin with morphological and immunohistochemical characteristics of a diffuse large B-cell lymphoma (as in WHO classification) with a high malignant potential. The immunophenotyping showed positive staining, diffusely for LCA, CD19, CD20, CD79 and focally for CD3, CD5, CD68, and no staining for epithelial and neuroendocrine markers or melanoma-specific markers (Figures 3, 4, and 5). A fine needle aspiration of one of the inguinal masses and biopsy of the bladder also revealed atypical lymphocytes (B-cells) consistent with diffuse large B-cell lymphoma. Staining was negative for acid-fast bacilli, while culture for bacteria and fungi did not yield any growth. The bone marrow biopsy did not show any infiltration by lymphoma. The patient was treated with systemic chemotherapy. Six cycles of CHOP (cyclophosphamide, hydroxydaunorubicin, oncovin, prednisone) were given resulting in healing of the penile ulcer and resolution of the inguinal lymphadenopathy. In the posttreatment transabdominal US the bladder and prostate lesions also had completely resolved (Figure 2(c)).

## 3. Discussion 

Primary penile lymphoma is extremely rare and in most of the reported cases it is located in the shaft of the penis and/or the glans penis [5]. Diffuse large cell lymphoma is the most common histological subtype [6]. Both B-cell and T-cell subtypes have been reported [7]. The clinical presentation of penile lymphoma includes indurated plaques, nodules, diffuse penile swelling, and ulceration with or without induration [6]. Rare associated symptoms include phimosis, priapism, lymphadenopathy, and testicular enlargement [8]. In most of the reported cases the disease has reached an advanced stage at the time of diagnosis [3]. The diagnosis of penile lymphoma can be demanding. A major differential diagnostic challenge is the distinction between this entity and the squamous cell carcinoma of the penis. In fact, failure to recognize lymphoma may result in unnecessary penile amputation, which is a common treatment option for squamous cell carcinoma and other malignant primary tumors of the penis, especially when the tumor involves the entire shaft. Therefore, a full physical examination and an excision biopsy are essential to reach the correct diagnosis. The differential diagnosis also includes vasculitis, trauma, and sexually transmitted diseases [9]. Since staging and differentiation between primary lymphoma and disease secondary to systemic lymphoma are important interims of treatment and prognosis, systemic radiological investigation, including CT, MRI, or positron emission tomography scan, should be undertaken [3]. While systemic chemotherapy is the treatment of choice for a secondary presentation of lymphoma in the penis, in primary penile lymphoma, treatment guidelines are difficult to establish, because of the very limited number of cases. Recommendations have included chemotherapy, local radiotherapy, surgery, or combined modalities of both surgery and chemotherapy or radiotherapy and chemotherapy [4–10]. Whether to use one, two, or all of these modalities to eradicate the tumor, while attempting to preserve the anatomy and physiology of the penis, is controversial. On reviewing the literature, it appears that the management of primary penile lymphoma depends mainly on the stage of the disease, as well as the age and performance status of the patient. Since malignant lymphoma is a systemic disease that can metastasize haematogenously, and treatment by chemotherapy and radiotherapy allows preservation of function of the penis, radical surgery appears to be contraindicated and should only be used after failure of other modalities [10]. Radiotherapy alone offers a potential cure rate of 65% in advanced stage lymphoma and thus could be considered as a valuable treatment option [3]. However, similar to surgery, radiotherapy may result in disfigurement or loss of erectile function. Moreover, there is no consensus as to the dose of radiotherapy and most importantly radiotherapy cannot eliminate occult disseminated disease. Systemic chemotherapy is a good treatment option because it preserves the erectile function and avoids disfigurement. The reported 2-year disease-free survival after chemotherapy has been as high as 83% for primary diffuse large cell lymphoma [11]. The usual chemotherapy regimen is CHOP; however other regimens have also been used, such as rituximab-containing ones [8]. Combined modality treatment seems to be the ideal treatment option, although toxicity can restrict the use of chemotherapy [12]. 

## 4. Conclusions

We report the case of an elderly patient with a penile ulcer that was associated with a diffuse large B-cell lymphoma infiltrating the adjacent organs. The tumour resolved completely after systemic chemotherapy with the CHOP regimen. Because of the rarity of this pathological entity, a high index of suspicion is required to correctly diagnose it and to institute appropriate treatment avoiding potentially unnecessary mutilating surgery.
